# Exploring Piezoelectric Actuation towards Its Applications in Laser Powder Bed Fusion Additive Manufacturing

**DOI:** 10.3390/s24123704

**Published:** 2024-06-07

**Authors:** Connor Griffin, Hanfei Mei, Sivaji Karna, Tianyu Zhang, Victor Giurgiutiu, Lang Yuan

**Affiliations:** Department of Mechanical Engineering, University of South Carolina, Columbia, SC 29208, USAskarna@email.sc.edu (S.K.); tz5@email.sc.edu (T.Z.); victorg@sc.edu (V.G.)

**Keywords:** additive manufacturing, piezoelectric actuation, laser powder bed fusion, ultrasound transmission, melt pool morphology

## Abstract

Piezoelectric materials, which exhibit a charge distribution across the surfaces in reaction to mechanical strain, find significant utility in actuation and sensing applications. Apart from actuation applications like acoustic devices, motors, and vibration damping, an emerging domain for ultrasonic actuators lies in additive manufacturing processes. Ultrasonic waves applied during solidification aim to modulate grain structure and minimize defects. This research focuses on a fixture designed to facilitate and optimize ultrasonic wave propagation through the build plate in laser powder bed fusion additive manufacturing by utilizing a piezoelectric transducer. Three implementations of piezoelectric transducers were evaluated based on their out-of-plane ultrasonic velocity transmissions. It was determined that a thin plate adhered to the surface of the piezoelectric transducer yielded the most favorable outcomes for implementation, achieving 100% transmission of velocity and energy. Preliminary analysis of melt pool morphology and defects in single-track laser scanning experiments demonstrated the impact of ultrasound on solidification, hinting at a novel approach to enhancing the printability of alloys in laser powder bed fusion additive manufacturing processes. The optimal fixture and the explored transducing efficiency could further guide advanced ultrasound testing to enable in situ defect and texture detection during the additive manufacturing processes.

## 1. Introduction

The motivation for this work lies in the need to improve the additive manufacturing (AM) of advanced metal parts. One of the inherent features in AM of high-strength alloys is the tendency to form columnar grains due to the rapid solidification with a high thermal gradient [1,2,3,4]. Columnar grain structures lead to anisotropy in mechanical properties [5]. Altering the microstructure from columnar grains to equiaxed grains is one of the fundamental requirements to control the microstructure fully and to achieve the desired properties in AM [6,7,8]. In addition, refining grains has been an effective means to eliminate solidification cracking for difficult-to-print alloys, such as high gramma prime Nickel-based superalloys and high-strength aluminum alloys [9,10,11,12,13]. The premise of the research is that the solidification process can be disrupted through the application of a high-power ultrasonic wavefield, thus resulting in the modification of melt pool dynamics and microstructures, including grains and defects [14,15].

Piezoelectric actuation makes use of the converse piezoelectric effect, where electrical energy is converted into mechanical energy. Usually, the displacement induced by a piezoelectric actuator remains minimal, typically ranging from 1 to 100 μm [16]. Piezoelectric materials, adept at converting electricity into vibrations, find widespread application in acoustic device technologies such as speakers, microphones, and earbuds. Moreover, the converse piezoelectric effect is frequently employed alongside the direct piezoelectric effect to generate and detect acoustic signals essential for sonar applications [16].

Piezoelectric materials find yet another application in piezoelectric motors, capable of generating an extensive array of movements, enabling precise positioning. These motors, operating on a millimeter scale, in conjunction with micro-electrical mechanical systems, can replicate the locomotion of insect-like robots [16]. Multilayer ceramic actuators, a type of piezoelectric actuator, have made their way into various consumer products, including office equipment and video cameras. For example, Canon’s cameras employ ultrasonic motors for lens movement, and similar technology has been integrated into inkjet printers [10]. In addition, Toyota has integrated actuators into electronically controlled suspension systems, while a novel injection system for diesel engines has been developed utilizing multilayer actuators [17].

A new application of piezoelectric actuation has come in the metal-based AM processes [14,18]. Research is being conducted on ultrasonic transducers to alter the grain structure and properties of additively manufactured parts. Hu et al. [14] introduced ultrasonic vibration using a piezoelectric transducer into the laser engineering net-shaping (LENS) process, one type of the Directed Energy Deposition (DED) AM processes, to reduce the grain size and microcracks. The ultrasonic vibration creates acoustic streaming and cavitation of the melt pool, leading to mixing and homogenization of the material and creating a more refined microstructure with reduced microcracks via promoting nucleation in the additively manufactured specimen. An ultrasonic vibration unit was used to convert electrical energy from the ultrasonic power generator into ultrasonic vibration using the piezoelectric converter. This ultrasonic vibration was then transmitted to the surface and, thus, the build plate using an acoustic transformer. The ultrasonic frequency for the testing was fixed at 41 kHz, and the ultrasonic amplitude was fixed at 5 μm [19]. Todaro et al. [14] implemented ultrasonics into a similar DED process for 316L stainless steel to increase the density of equiaxed grains. The ultrasound equipment used consisted of a Sonic systems L500, 20 kHz, 500 W power ultrasound processor, and an ultrasound sonotrode made from 4140 steel with a 25 mm diameter and 30 μm amplitude of displacement. A frequency of 20 kHz was chosen for their study due to the threshold of cavitation increasing at a rapid rate above 20 kHz, the maximum power output being compromised at higher frequencies, and noise increasing at frequencies below 16 kHz [14]. Todaro et al. [20] also explored the use of ultrasound during the DED of Ti-6Al-4V using a 20 kHz ultrasonic transducer with a vibration amplitude of 30 μm and reported the refinement of grain structure from columnar to equiaxed. Wang et al. [18] performed similar work to induce cavitation and acoustic streaming in the melt pool via changing the ultrasound frequency upon depositing Inconel 718. The ultrasonic vibration amplitude was around 5 μm in the vertical direction. Varying frequencies of 25 kHz, 33 kHz, and 41 kHz were used. The average grain size decreased with increasing frequency, while the porosity level was raised at higher frequencies. Zhang et al. [21] introduced ultrasonic vibration into the laser metal deposition process of Al-12Si alloy. Using a 1000 W power ultrasonic vibration along with a frequency of 20 kHz and an ultrasonic amplitude of 25 μm, they were able to increase the density and strength of the additively manufactured Al-12Si.

Laser powder bed fusion (LPBF) offers significantly higher spatial resolution compared to DED. Although promising grain refinement has been confirmed with DED, the ultrasound-assisted approach in LPBF is still in its very early stages of exploration. Only one piece of literature was reported by Yan et al. [15], who investigated ultrasonic vibration in LBPF of superalloy, GH5188. An ultrasonic generator with a frequency of 40 kHz, power output of 60 W, and ultrasonic displacement of 20 μm was used in their study. In order to reduce powder accumulation in ultrasonic wave nodes, the power and amplitude in their study were optimized at 15 W and 2.5 μm.

Even though much research has been performed to study the application of piezoelectric material and the impact of ultrasound on melt pool behavior via AM, the detailed experimental setup for ultrasound generation and transducing efficiency has not been investigated in LPBF. Therefore, our study is highly exploratory, with the following goals: (a) Determine methods for integrating a high-power ultrasonic field into the LPBF process. (b) Build an experimental testbed featuring an adjustable-power ultrasonic transducer. (c) Investigate the transmission of ultrasonic power across diverse interfaces, aiming to identify an optimal solution. (d) Characterize the product using LPBF with the optimized transmission rate. Beyond the scope of this work, ultrasound testing has been widely employed to analyze the porous structure, detect internal defects such as voids and cracks, as well as for the analysis of material properties such as density, material strength, and Young’s modulus [22,23,24]. Recent progress has enabled ultrasound testing to reveal mesoscale crystalline textures for LFBF samples [25,26]. As an extension, the outcomes of this study can further guide the integration of ultrasound sensors for in situ nondestructive testing in AM processes, utilizing piezoelectric transducers with higher frequencies for detecting defects, properties, and microstructures.

## 2. Experiment Method

### 2.1. Ultrasound Testbed Preparation

**Figure 1:**
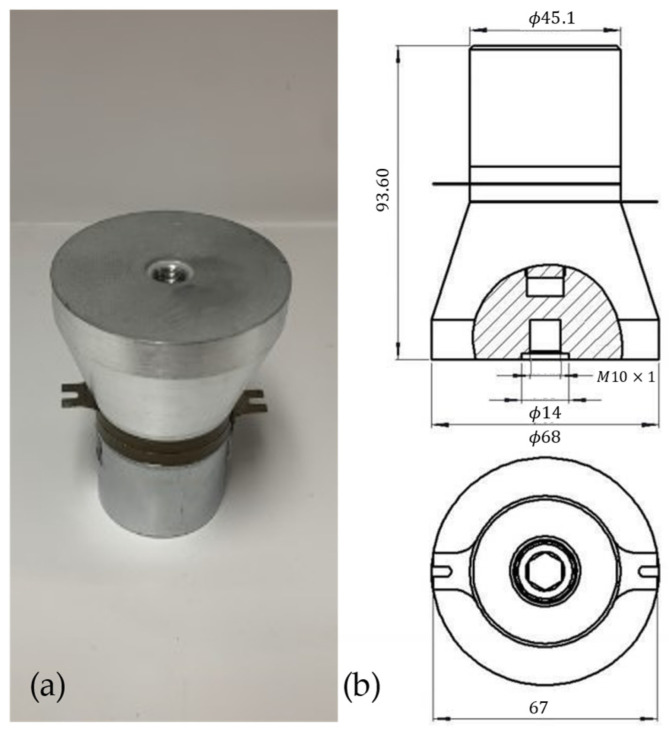
(**a**) Image of the bolt-clamped transducer. (**b**) Dimensions of the bolt-clamped transducer in millimeters.

**Figure 2:**
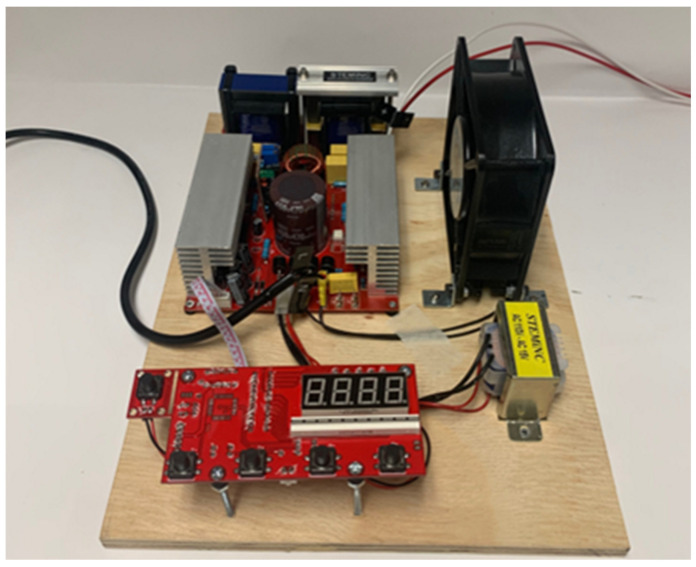
Ultrasonic power generator.

Similar to the approaches discussed by Hu et al. [5], Todaro et al. [6,7], and Wang et al. [8], the implementation of ultrasonic vibration by means of a piezoelectric transducer in the LPBF process was investigated. The ultrasonic equipment used consisted of a 100 W power, 20 kHz resonant frequency bolt that was clamped. Langevin piezoelectric transducer manufactured by STEMiNC (SMBLTF20W120, Davenport, FL, USA), pictured in Figure 1. Ultrasonic vibration of the transducer surface occurs on the wider end of the transducer, as can be seen at the top in Figure 1. To actuate the piezoelectric transducer, a 200 W adjustable frequency power generator from STEMiNC (SMUG200W2068ND, Davenport, FL, USA) was used. Power from the generator was capable of being tuned in intervals of 20 W from 0 to 200 W. The maximum power output used in this case was 100 W, corresponding to the maximum power output of the transducer. The frequency was capable of being tuned from 10 kHz to 40 kHz and, in this case, was fixed at 20 kHz for testing, as this was the resonant frequency of the transducer and, in previous studies, was responsible for the best results [14]. The ultrasonic power generator is shown in the top left of Figure 2. The bottom left is the LED control panel, where power output was specified. On the top right is the cooling fan, and on the bottom right is the AC transformer.

**Figure 3:**
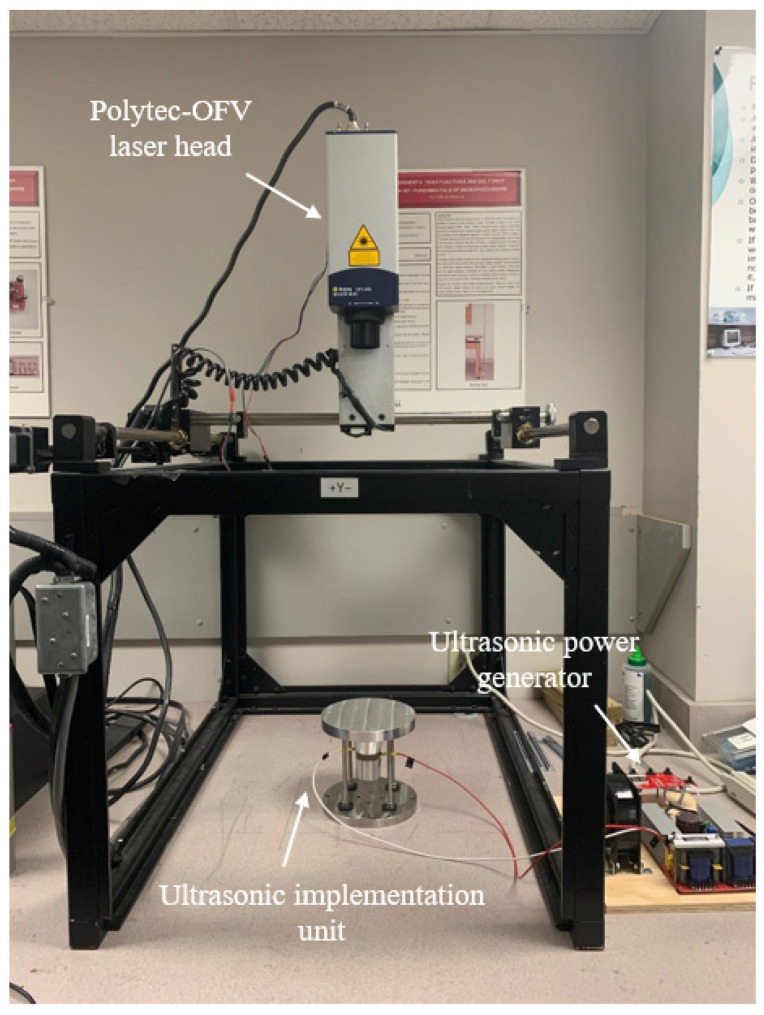
Experimental setup for ultrasonic velocity measurement.

To quantify ultrasonic vibration magnitudes, a scanning laser Doppler vibrometer (SLDV) was used to measure the transducer’s out-of-plane ultrasonic velocity and the AM build plate surfaces. The Polytec-OFV 501 SLDV (Polytec, Baden-Württemberg, Germany), shown in Figure 3, has a frequency range of 1.5 MHz. The SLDV was used to take point measurements of the out-of-plane ultrasonic velocity of the surfaces across various linear paths and increments. The transducer was placed on styrofoam while it was measured using the SLDV in order to prevent the transducer from rattling.

Super glue is utilized to connect the transducer with the thin aluminum plate. The surface preparation (abrading with 600-grit sandpaper, cleaning using degreaser and cloth gauze, applying acid, and cleaning) was performed to ensure an ideal bond between surfaces that were free of dirt and debris. After the superglue was applied to the surfaces and left to cure for 24 h, a 500 g mass was placed on top of the plate and the transducer to ensure constant contact between the surfaces and a good bond.

**Figure 4:**
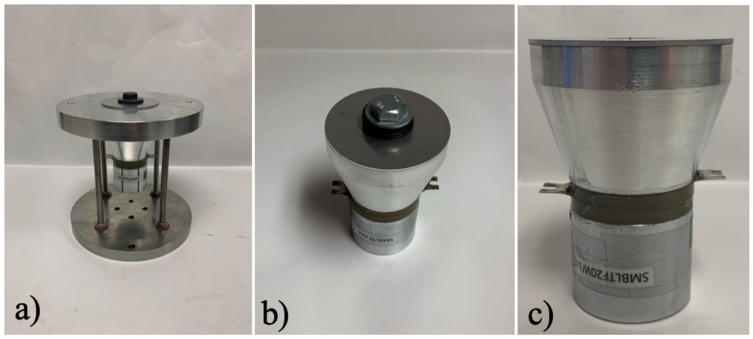
Images of ultrasonic implementation setups. (**a**) Transducer connected to the aluminum build plate suspended by four rods with a thin plate connected to its surface via a bolt. (**b**) A thin plate securely bolted to transducer surface. (**c**) A thin plate superglued to the transducer surface.

Three different ultrasonic transducing setups were applied to the LPBF process: 1. an aluminum 6061-build plate suspended by four rods with a thin plate connected to its surface via a bolt; 2. a thin plate securely bolted to the surface of the transducer; and 3. a thin plate glued to the transducer surface; all setups are pictured in Figure 4. For transducer implementations where a thin plate is bolted to the surface, ultrasonic gel was used between metal boundaries to improve ultrasonic velocity transmission between surfaces. The three implementations progressively increase in complexity regarding the integration of the ultrasound transducer with the build platform and modification of the LPBF system. Utilizing a bolt facilitates the assembly and removal of the build platform. However, transitioning to a reduced build platform, equivalent to the size of the transducer’s top surface, necessitates modifications to the recoating system to accommodate the smaller powder recoating area.

### 2.2. Processing of SLDV Measurements

Due to the variations and inaccuracies in the frequency spectrum and velocity measurements, the out-of-plane displacement of the build surface could not be perfectly measured. Instead, in this study, it was found that the best way to quantify the rate of ultrasonic vibration transmission from the ultrasonic transducer through to the build plate is by calculating the root mean square (RMS) of the out-of-plane surface velocity, as shown in Equation (1). Utilizing RMS of the velocity allows for the ability to keep the data in measured units, while also providing a correlation to energy input. The root mean square velocity formula is shown in Equation (1), where v is velocity and n is the number of measurements taken. As the RMS is a measurement of velocity, it allows for a relation to the kinetic energy transmitted to the build plate to be made, as shown in the definition of the kinetic energy in Equation (2). The displacement calculation is shown in Equation (3), where v(t) is the out-of-plane velocity, f is peak frequency of the frequency spectrum, and $e^{-i\frac{\pi }{2}}$ is the phase difference.

(1) $$MS=\sqrt{\frac{1}{n}\sum_{i}v_{i}^{2}}$$

(2) $$KE=\frac{1}{2}mv^{2}$$

(3) $$u\left(t\right)=\frac{v(t)}{2\pi f}e^{-i\frac{\pi }{2}}$$

In order to quantify the effectiveness of the ultrasonic transducer setup, a rate of transmission parameter was used. Transmission rates for the out-of-plane surface velocity were computed as a percentage of the RMS velocity, as well as a percentage of the energy, shown in Equations (4) and (5), respectively. RMS_measured_ represents the RMS of the out-of-plane velocity of the thin plate surface, while RMS_transducer surface_ represents the RMS of the out-of-plane velocity of the pristine transducer surface. As a circular transducer, the ultrasonic velocity measured was consistent across radial paths, regardless of the path. Four paths were tested to ensure the repeatability of this experiment.

(4) $$\%\;RMS\;transmission=\frac{{RMS}_{measured}}{{RMS}_{transducer\;surface}}\times 100$$

(5) $$\%\;Energy\;transmission=\frac{{RMS}_{measured}^{2}}{{RMS}_{transducer\;surface}^{2}}\times 100$$

## 3. Result and Analysis

### 3.1. Results for the Single Ultrasonic Transducer

**Figure 5:**
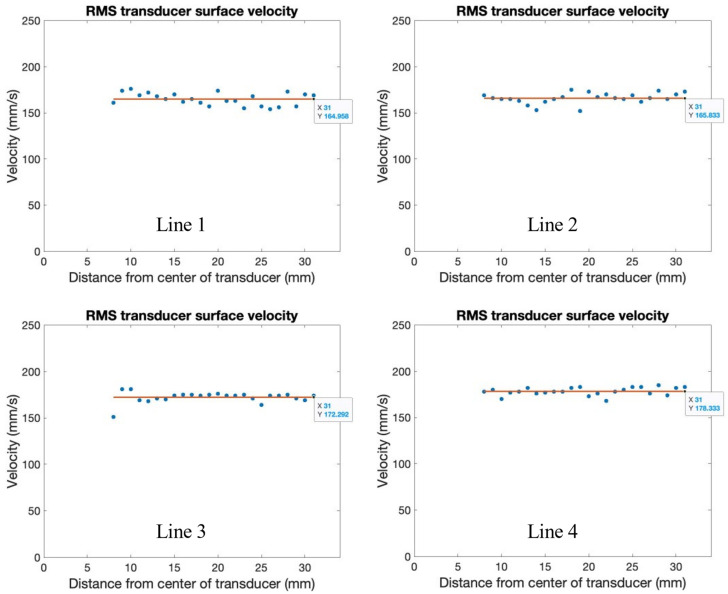
Measurement of transducer surface RMS velocity across radial paths.

Across radial paths, the measurements of RMS velocity are seen to be consistent, regardless of the distance to the center of the transducer, pictured in Figure 5, with lines shown with the average RMS velocity along each radial path. The maximum RMS velocity occurred on radial path four, with a value of about 178 mm/s, and the minimum RMS velocity occurred on radial path one, with a value of about 164 mm/s. The variation in the measured RMS velocity can be attributed to the memory limitations of the Polytec-OFV SLDV, where the maximum time-frame measurement was 80 ms. Due to the beat phenomena, variation in the number of peaks or troughs of the waveform captured can cause variations in the overall measured RMS velocity. If more time is allowed for the RMS velocity to be measured, more beats will be captured, and a convergence of the RMS velocity along radial paths will occur. In calculating the rate of transmission for the setups evaluated for ultrasonic implementation, the RMS velocity of radial path 4 was used as the RMStransducer surface, as it presented the maximum value.

**Figure 6:**
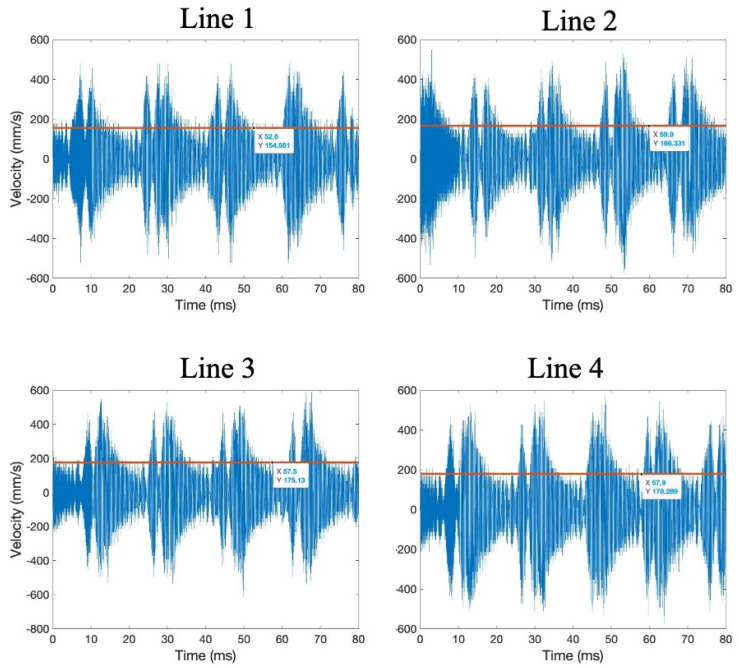
Out-of-plane surface velocity waveforms across radial paths.

The waveforms and RMS velocity of the transducer surface at points about 23 mm from the center of the transducer were also taken and compared, shown in Figure 6. These point measurements and comparisons of waveform shapes and velocities provide a better comparison to confirm uniform transducer output. In analyzing the transducer velocity at similar points from the center of the transducer, it is seen that the same beat phenomenon occurs across all radial paths, and that all waveforms have similar maximum and minimum out-of-plane velocities of around 500 mm/s. The measurements taken for the out-of-plane transducer surface velocity serve as a baseline for comparing maximum wave transmission through the build fixture or thin plate and to the build plate.

### 3.2. Results for Build Plate Bolted onto the Ultrasonic Transducer

The ultrasonic setup, consisting of a transducer connected to an aluminum build plate suspended by four rods with a thin plate connected to its surface with a bolt, is shown in Figure 4a. The aluminum build plate is supported via four metal rods securely screwed to both the top aluminum plate and bottom stainless-steel plate. Washers were placed between the securing nuts to reduce rattle and unnecessary vibration and energy loss in the system. The thin plate is bolted through the top plate, connected straight through to the transducer, with a flat metal washer and a rubber washer between the bolt head and the thin plate.

**Figure 7:**
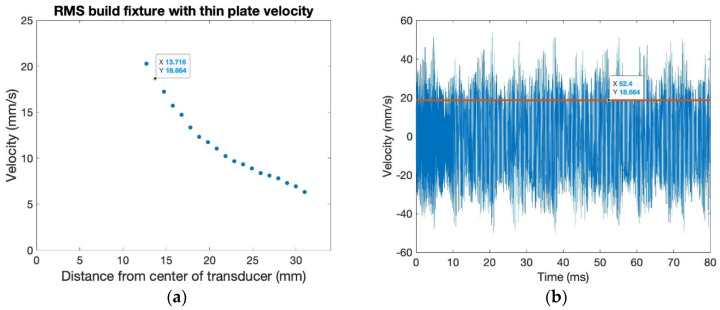
Build fixture with the thin plate: (**a**) RMS velocity across the radius, and (**b**) velocity waveform at 14 mm from the center of the transducer.

The variation in the RMS velocity of the thin plate bolted to the build plate surface decreases as radial distance increases, with maximum velocity occurring towards the center of the thin plate, where it is bolted to the build fixture, as shown in Figure 7a. The maximum RMS of out-of-plane ultrasonic surface velocity is around 21 mm/s. The RMS transmission rate for this ultrasonic setup is only around 12%, while the energy transmission rate is only around 1.4%. The out-of-plane velocity waveform of the thin plate is shown in Figure 7b. The velocity waveform does not have the same beat phenomena as that of the transducer surface, with the thin plate connected to the build fixture having a more uniform and consistent surface velocity. Explanations for low RMS and energy transmission rates include the need for the transmission through two metal boundaries: from the transducer to the build plate, and then from the build plate to the thin plate. Additional reasons include the attachment of the top aluminum plate to the bottom plate, leading to potential vibration being dampened from energy being used to vibrate the bottom plate.

The advantages of this assembly include that there is no need for dampening below the transducer, as it is affixed to the top build plate, as well as the fact that there is relative ease of assembly and removal of the transducer surface. Disadvantages include the transmission rate being much less than desirable, which may not be worth implementing, and the fact that the size of printing is limited in this setup due to the height of the suspended build plate.

### 3.3. Results for Thin Plate Bolted on the Ultrasonic Transducer

**Figure 8:**
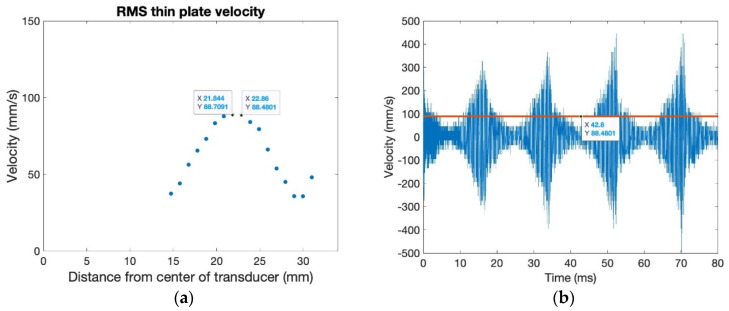
Thin plate connected with a bolt: (**a**) RMS velocity across the radius, and (**b**) bolt velocity waveform at 23 mm from the center of the transducer.

Figure 4b shows a thin plate connected to the ultrasonic transducer via a bolt. The thin plate is bolted to the transducer, with a flat metal washer and rubber washers between the bolt head and plate. The ultrasonic velocity, as it varies across the radial path, is pictured in Figure 8a for a thin plate connected to the transducer surface with a bolt. The RMS of the velocity of the thin plate bolted to the transducer peaks at around 88–89 mm/s at about 22–23 mm from the center of the transducer. Like the transducer’s output, the thin plate’s measured velocity was consistent across radial paths; thus, a comparison of only one path is necessary. The RMS transmission rate for this ultrasonic setup is around 50%, while the energy transmission rate is only around 25%. Better transmission rates occur with this ultrasonic implementation setup due to only one metal boundary for transmission, as well as the elimination of the bottom plate, reducing energy loss in the system.

The transducer surface velocity at a distance of about 23 mm from the center of the transducer is shown in Figure 8b. The beat phenomena occurring in the velocity waveform are different from that occurring on the surface of the transducer, with a sharp increase and then decrease in maximum amplitude occurring, followed by a short period of near zero out-of-plane surface velocity. Of note is that the maximum out-of-plane surface velocity measurement is similar to the maximum reached on the surface of a pristine transducer. Advantages of this setup include the extreme ease of assembly and disassembly and the achievement of similar maximum ultrasonic velocities. Disadvantages include limitations in printing due to the bolt head sticking up from the surface as well as the length of maximum ultrasonic transmission, limiting printing area for maximum efficacy of ultrasonic motion in the AM process.

### 3.4. Results for Thin Plate Glued to the Ultrasonic Transducer

**Figure 9:**
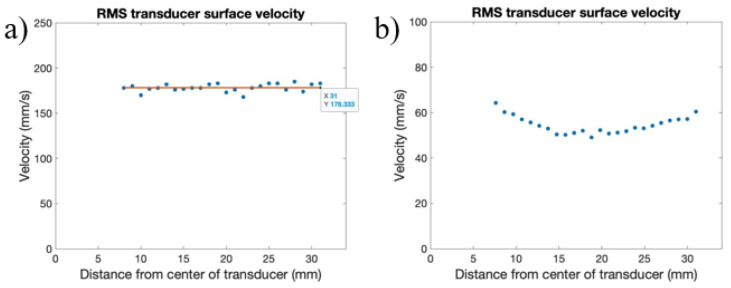
Transducer surface velocity across radius for the (**a**) pristine transducer and (**b**) “tired” transducer.

**Figure 10:**
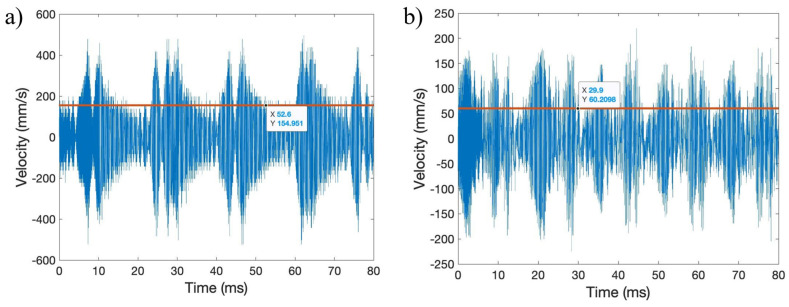
Out-of-plane surface velocity waveform for a (**a**) pristine and (**b**) “tired” transducer.

A thin plate connected to the ultrasonic transducer surface using superglue is shown in Figure 4c. Measurements for a thin plate affixed to the transducer surface using superglue were initially conducted with a tired transducer, with the output shown in Figure 9 and Figure 10. The out-of-plane velocity of the transducer drastically decreases, and is no longer consistent, as it varies along radial distance from the center of the transducer. As shown in Figure 9, the RMS of out-of-place surface velocity is compared to that of a tired and pristine transducer. The shape of the ultrasonic vibration waveforms changes, with an irregular beat phenomenon forming with a tired transducer compared to that of a pristine transducer, as shown in Figure 10.

The rate of ultrasonic transmission for a thin plate superglued to the transducer surface is about 100% for both the RMS transmission rate as well as the energy transmission rate. Maximum transmission rate values occur toward the center and edges of the thin plate, while the minimum ultrasonic transmission rates occur at about half the radius of the transducer. The ultrasonic velocity waveform for the superglued thin plate has a similar beat pattern as those that occur with a tired transducer. The maximum out-of-plane surface velocity of the superglued thin plate also produces similar values to those of a tired transducer.

**Figure 11:**
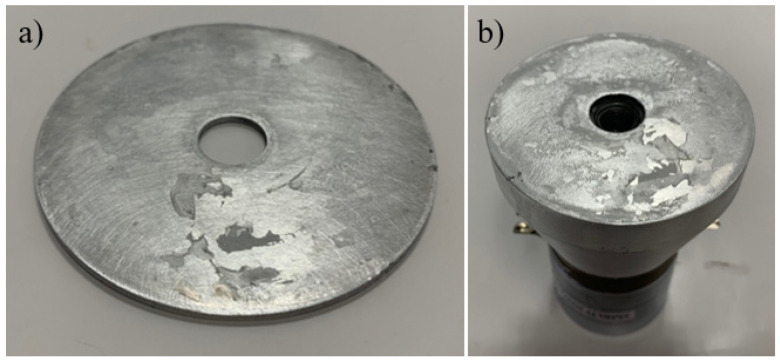
Super glue left over on the (**a**) thin plate and (**b**) transducer surface after removal.

The advantages associated with a thin plate superglued to the transducer surface, other than its superior transmissibility compared to the other ultrasonic setups, include the ease of assembly and removal of the thin plate. The superglued plate can be pried off the transducer surface very easily. After the removal of the thin plate, the residue of super glue is left behind. This super glue residue can easily be removed with the use of isopropyl alcohol, a degreaser, and an exacto scraper. The transducer and thin-plate surfaces after the removal of super glue residue are shown in Figure 11. After removal, the transducer and thin plate can be prepared in the same way discussed earlier in order to be superglued together and re-used. Another advantage is the ability to build multiple layers continuously on the build surface without the bolt head, preventing new powder deposition on top of the build surface. A disadvantage is the time between consecutive tests using this method. In order to run consecutive tests using the same transducer, a minimum of 24 h is needed between prints to allow the super glue between the thin plate and transducer surface to cure.

### 3.5. Preliminary Metallographical Results

The thin plate, made of aluminum alloy AA6061, which was superglued to the transducer surface, was placed on the build platform to preliminarily explore the impact of ultrasound on the melt pool behaviors during the laser scanning process with the LPBF printer (Aconity3D MIDI, Herzogenrath, Germany). The selection of this setup is due to the best RMS and energy transmission rates among other setups examined above. Via adjusting the height of the original build platform, the top of the thin plate was aligned with the focal plane of the laser. Similar to the laser-welding process, we laser-scanned single tracks on the top surface of the thin plate with different process parameters with and without ultrasound. After finishing the scanning, the thin plate was sectioned perpendicular to the scanning direction. The cross-sections of the laser tracks were grind, polished, and etched to reveal the melt pool morphology. Additional material characterizations using scanning electron microscopy (SEM) were also carried out to examine any potential impacts of ultrasound on the microstructures.

**Figure 12:**
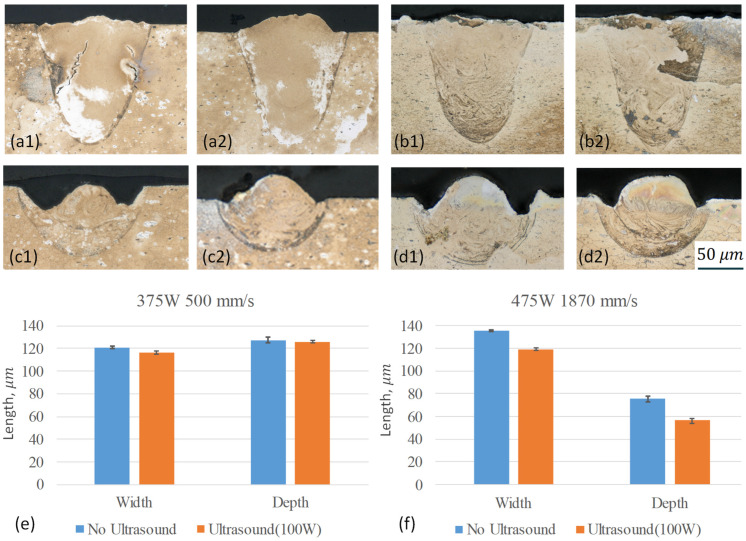
Comparison of melt pool morphology with and without ultrasound under two different process conditions. Laser power of 375 W, speed of 500 mm/s: (**a1**,**b1**) without ultrasound, (**a2**,**b2**) with ultrasound. Laser power of 450 W, speed of 1870 mm/s: (**c1**,**d1**) without ultrasound, (**c2**,**d2**) with ultrasound; (**e**,**f**) melt pool size comparison.

Figure 12 shows the melt pool under two distinct process conditions, which yield energy densities and corresponding melt pool morphologies: (1) laser power 375 W and speed 500 mm/s, and (2) laser power 450 W and speed1870 mm/s. To potentially reduce the impacts of the process variations, two tracks were conducted for each condition. Without ultrasound, the first condition generated a keyhole type of melt pool, where the depth of the melt pool is significantly larger than the width. In contrast, a conductive melt pool was formed with the second condition, where the melt pool width exceeded the depth. Upon comparing the repeated tracks, similar melt pool morphologies were observed, suggesting the consistency of the laser-scanning process.

The melt pool morphology is consistent with the linear energy output to the plate, where the first condition (0.75 J/mm) is more than three times higher energy density than that of the second condition (0.24 J/mm). With ultrasound running at 100 W and 20 kHz, the mode of the melt pool did not change for each condition. Figure 12e,f presents the mean melt pool width and depth with the standard derivations. The melt pool width and depth were both reduced in comparison to the cases without ultrasound. The reductions are approximately 3.7% for the width and 1.2% for the depth, with the high energy density case. This value increases to 12.2% and 24.5%, with the lower energy density case for the width and depth, respectively. The potential reduction in melt pool size could be hypothetically attributed to the enhanced mixing within the melt pool. As previously discussed in the literature [15,19], ultrasound-assisted processing operating at a frequency of 20 kHz has been shown to induce acoustic streaming, cavitation, and mechanical vibration within the melt pool. During the melting process, the Marangoni force, driven by surface tension due to temperature gradients in AA6061, typically induces a downward flow aimed at deepening the melt pool by transporting hot melt from the surface to the bottom. The enhanced fluid flow induced using ultrasound may disrupt the downward Marangoni flow, consequently resulting in a reduction in melt pool size. With lower energy density, the surface tension gradient is expected to be smaller, thereby weakening the Marangoni force. This permits a more significant influence from ultrasound compared to cases with higher energy density.

**Figure 13:**
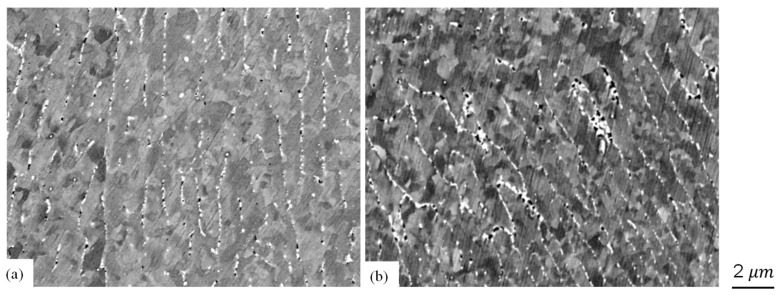
Comparison of microstructure for the case with laser power of 375 W, speed of 500 mm/s: (**a**) without ultrasound, (**b**) with ultrasound.

In addition to the melt pool morphology, an apparent reduction in microcracks was observed for the high energy density case (Figure 12(a1,a2)). Furthermore, Figure 13 illustrates the grain structures and secondary phase presence with and without ultrasound under the condition of laser power at 376 W and a speed of 500 mm/s. Generally, a reduction in subgrain size and spacing between the accumulated secondary phases can be observed. These observations align with findings reported in the literature regarding the DED [14] and LPBF [15] processes. Given the focus of this study on examining various ultrasound configurations, more in-depth characterizations are required to explain ultrasound’s impact on melt pool dynamics, as well as the microstructure in the LPBF process further. Nevertheless, this exploration suggests the potential role of ultrasound in AM processing.

## 4. Summary, Conclusions, and Further Work

### 4.1. Summary

The application of piezoelectric actuation has been reviewed, with an emphasis on its application in the additive manufacturing process. Methods for the introduction of ultrasonic waves in the additive manufacturing process through the use of a piezoelectric transducer have been introduced, and their effectiveness in ultrasonic transmission to the build plate has been analyzed, with the final results pictured in Table 1.

The three methods for ultrasonic implementation analyzed include a transducer connected to an aluminum build plate suspended by four rods with a thin plate connected to its surface via a bolt, a thin plate securely bolted to the surface of the transducer, and a thin plate glued to the transducer surface. The methods of ultrasonic implementation via a piezoelectric transducer were analyzed based on their ease of assembly/removal, and the RMS transmission rate of the out-of-plane ultrasonic velocity and energy. Testing for a transducer connected to the aluminum build plate suspended by four rods with a thin plate connected to its surface via a bolt produced a transmission rate of 12% of RMS velocity and only around 1.4% of energy transmission. For a thin plate securely bolted to the surface of the transducer, the RMS transmission rate is around 50%, while the energy transmission rate is only around 25%.

### 4.2. Conclusions

The best transmission rate, and the method of ultrasonic implementation that would prove to be the most useful in application in the AM process, occurred in the thin plate glued to the transducer surface. The transmission rates were close to 100% for both the RMS transmission rate and the energy transmission rate. Maximum transmission rate values occur toward the center and edges of the thin plate, and minimum rates occur at about half the radius of the transducer. The variation of the melt pool size and the reduction in cracks by applying ultrasound in this study suggests potentially a novel approach to improve the solidification microstructure with the elimination of defects in the LPBF process.

### 4.3. Suggestions for Further Work

Suggestions for future work include exploring the implementation of ultrasonic vibration into the AM process with transducers capable of increasing power output. In the evaluated case, the maximum power output of the ultrasonic transducer was only 100 W, but the power generation unit used was capable of 200 W. In addition to the exploration of ultrasonic vibration transmission with increased power output, investigation can be conducted into the ultrasonic transmission with transducers of different resonant frequencies. In the current case, the power generation unit was capable of being tuned from 20 kHz to 68 kHz, but only a 20 kHz resonant frequency ultrasonic transducer was evaluated.

The preliminary tests in the LPBF system have only been conducted for bare plates with line scans. Unambiguous evidence proves the impacts of ultrasound on the melt pool size and microcracks. A detailed microstructure analysis, including grain texture and composition quantification, needs to be performed to confirm the impact of ultrasound in AM processing further. To go one step further, explorational experiments need to be performed on actual powder before being demonstrated on a component level.

## Figures and Tables

**Table 1:** RMS and energy transmission rates for all ultrasonic setups evaluated.

Ultrasound Implementation Method	RMS Transmission	Energy Transmission
Thin plate connected to build plate surface via bolt	12%	1.4%
Thin plate connected to transducer surface via bolt	50%	25%
Thin plate glued to transducer surface	100%	100%

## Data Availability

Data are contained within the article.
